# Disgust sensitivity in early pregnancy as a response to high pathogen risk

**DOI:** 10.3389/fpsyg.2023.1015927

**Published:** 2023-02-27

**Authors:** Šárka Kaňková, Lea Takács, Jana Hlaváčová, Pavel Calda, Catherine Monk, Jan Havlíček

**Affiliations:** ^1^Department of Philosophy and History of Science, Faculty of Science, Charles University, Prague, Czechia; ^2^Department of Psychology, Faculty of Arts, Charles University, Prague, Czechia; ^3^Fetal Medicine Centre, Department of Obstetrics and Gynecology, First Faculty of Medicine, Charles University and General University Hospital, Prague, Czechia; ^4^Department of Obstetrics and Gynecology, Columbia University Medical Center, New York, NY, United States; ^5^Division of Behavioral Medicine, New York State Psychiatric Institute, New York, NY, United States; ^6^Department of Zoology, Faculty of Science, Charles University, Prague, Czechia

**Keywords:** COVID-19 pandemic, disgust, behavioral immune system, compensatory prophylaxis hypothesis, pregnancy, age, nausea and vomiting in pregnancy

## Abstract

**Introduction:**

Considered a part of the behavioral immune system (BIS), disgust sensitivity is expected to be adjusting as a response to the actual level of the environmental health risks.

**Methods:**

In this preregistered study, we tested the hypothesis that disgust sensitivity would be higher during the COVID-19 pandemic compared to the pre-pandemic period in pregnant women. In this between-subject study with a longitudinal trend design, we administered the Disgust Scale-Revised to 200 pregnant women before the pandemic and to 350 pregnant women during the pandemic.

**Results:**

We found a small but significant effect of the pandemic on disgust sensitivity, such that higher disgust sensitivity was found in women pregnant during the pandemic. This effect was stronger in primiparae, however, the interaction between parity and the pandemic period was not significant. Disgust sensitivity decreased with age. No differences in terms of nausea and vomiting were found between the women pregnant before and during the pandemic.

**Discussion:**

Our findings indicate that although BIS is presumed to function as a complex mechanism to prevent health-threatening behaviors, its activation in pregnant women during the COVID-19 pandemic is rather weak.

## Introduction

To protect themselves from various pathogens posing potential health risks, vertebrates including humans evolved physical and physiological barriers and mechanisms constituting a complex immune system (Abbas et al., 2014). Moreover, apart from those physiological defenses, individuals are also equipped with a set of psychological mechanisms referred to as the behavioral immune system (BIS), which helps to minimalize the risk of disease by detecting and avoiding health-threatening substances (Schaller et al., 2007). It is assumed that the BIS operates mainly through the experience of disgust that is elicited by the disease-causing substances (Curtis et al., 2004; Oaten et al., 2009).

Previous research has shown that there is indeed an association between higher disgust sensitivity and a lower risk of contracting an infection (Stevenson et al., 2009), presumably due to enhanced behavioral avoidance of disease-bearing sources. Similarly, a recent study (Cepon-Robins et al., 2021) reported a negative association between pathogen disgust sensitivity and biomarkers of immune response to viral and bacterial infection in Ecuadorian Shuar communities living in a high-pathogen environment.

In general, there is high inter-and intra-individual variation in disgust sensitivity. To explain this variation, Fessler et al. (2005) proposed the Compensatory Prophylaxis Hypothesis (CPH), claiming that disgust sensitivity adjusts adaptively depending on one’s immunocompetence. CPH was originally developed in the context of changes in progesterone levels during the menstrual cycle, since progesterone is thought to have immunosuppressive effects (Fessler and Navarrete, 2003). Therefore, in the luteal phase, when progesterone levels are highest, disgust sensitivity should also be increased to compensate for the immunosuppression. However, research has not supported this assumption unambiguously. While some studies found a positive correlation between disgust sensitivity and progesterone levels (Fleischman and Fessler, 2011; Żelaźniewicz et al., 2016; Miłkowska et al., 2021a), others found no such association (Jones et al., 2018; Timmers et al., 2018; Stern and Shiramizu, 2022).

Further research testing the CPH examined whether individuals who are more vulnerable to infectious diseases display higher disgust sensitivity. Again, no unequivocal evidence for this assumption has been found. Whereas some studies reported a significant association between disgust sensitivity and current health status (Miłkowska et al., 2019), others failed to do so (de Barra et al., 2014; Oaten et al., 2017). Interestingly, Miller and Maner (2011) found that recent illness increased attention to and avoidance of disfigured faces, which were considered an indication of pathogens. However, a direct replication of this study did not confirm the association between a recent illness and attention paid to disfigured faces (Tybur et al., 2020).

Apart from the inter-and intra-individual variability in terms of vulnerability to infection, disgust sensitivity might also vary across the population depending on the actual level of environmental risks. The recent COVID-19 pandemic represents a situation that elicits an extremely high pathogen risk, and it can be therefore expected that populations affected by the pandemic would show a generally higher disgust sensitivity. Some studies have indeed shown that during the COVID-19 lockdown, the disgust sensitivity was higher compared to the period before the pandemic in different samples, including students (Stevenson et al., 2021) and reproductive-age women (Miłkowska et al., 2021b).

Higher activation of BIS can be expected during pregnancy, which is considered a vulnerable period from the immunological point of view. On the one hand, there are complex suppressive processes to tolerate the semi-allogeneic fetus, on the other hand, the maternal immune system adapts to protect the developing fetus effectively against infections (Abu-Raya et al., 2020). We may expect an increase in disgust sensitivity especially during the first trimester of pregnancy (Fessler et al., 2005; Żelaźniewicz and Pawłowski, 2015; Kaňková et al., 2022), as maternal infection may result in severe fetal morbidity at this period. As the COVID-19 pandemic poses significant health-related risks, it can be assumed that women at an early stage of pregnancy during the COVID-19 pandemic would display a particularly large increase in disgust sensitivity as a response to the high pathogen risk.

The main aim of the present study was to assess the effects of the COVID-19 pandemic on disgust sensitivity in early pregnancy by comparing disgust sensitivity in women who were pregnant before and during the pandemic. According to CPH, disgust increases when the individual is at higher risk of contracting infection (Fessler et al., 2005). Therefore, we hypothesized that disgust sensitivity would be higher in women pregnant during the pandemic compared to those pregnant before the pandemic. Moreover, we hypothesized that there would be no significant differences in the frequency of nausea and vomiting in pregnant women before and during the pandemic. Although there are some similarities between NVP and disgust in pregnancy, for example, both NVP and disgust sensitivity tend to peak in the first trimester (Lacroix et al., 2000; Fessler et al., 2005; Żelaźniewicz and Pawłowski, 2015), the main aim of NVP is to protect the mother and the fetus against food containing potentially toxic abortifacients and teratogens (Hook, 1976; Profet, 1992, 1995; Flaxman and Sherman, 2000; Fiurašková et al., 2021), not against the risk of contracting infection. Therefore, we expect that unlike disgust, NVP will not be affected by the COVID-19 outbreak. We also hypothesized that there would be differences in disgust sensitivity depending on parity (i.e., differences between primiparous and multiparous women), but we did not formulate a specific hypothesis regarding this association as the results of the previous studies are inconsistent (Żelaźniewicz and Pawłowski, 2015; Prokop and Fancovicova, 2016). Our hypotheses were preregistered before launching the data collection during the pandemic period (OSF).^1^

## Materials and methods

### Procedures and participants

#### The sample recruited before the COVID-19 pandemic

Between November 2017 and November 2019, we recruited 205 pregnant women within the prospective cohort study assessing the effects of hormonal contraception on partner selection, relationship satisfaction, the likelihood of conception, and the frequency of nausea and vomiting in pregnancy. The women were recruited in collaboration with the General University Hospital in Prague (Dept. of Obstetrics and Gynecology) during their prenatal medical check-ups between the 11^th^ and 14^th^ gestational week. They were approached by the hospital staff and asked to complete a questionnaire focusing on their sociodemographic background and health status and the questionnaires related to disgust sensitivity (Disgust Scale-Revised; DS-R) and nausea and vomiting (Index of Nausea, Vomiting, and Retching; INVR). Only healthy women with natural conception (no assisted reproduction or hormonal treatment) were included in the study. Five women were excluded due to missing values on the DS-R (more than one-fifth of items for each subscale or the whole questionnaire unanswered). The final sample consisted of 200 pregnant women aged 19 to 44 years (see Table 1 for more details regarding the sample characteristics).

**Table 1:** Characteristics of the sample.

	Before the COVID-19 pandemic (*N* = 200)	During the COVID-19 pandemic (*N* = 350)
Length of pregnancy at the time of the study enrolment (days)		
Mean (SD)	89.7 (3.98)	89.1 (3.45)
Age (years)*		
Mean (SD)	30.7 (4.31)	31.9 (4.32)
Parity*		
Primipara, *N* (%)	120 (61.9)	177 (50.6)
Multipara, *N* (%)	74 (38.1)	173 (49.4)
Missing data	6	0
Smoking		
No, *N* (%)	174 (87.0)	310 (89.1)
Yes, *N* (%)	26 (13.0)	38 (10.9)
Missing data	0	2
Educational level		
Elementary school, *N* (%)	16 (8.1)	16 (4.6)
Secondary school, *N* (%)	61 (30.8)	94 (27.2)
University, *N* (%)	121 (61.1)	235 (68.1)
Missing data	2	5

*p < 0.05, **p < 0.01.

#### The sample recruited during the COVID-19 pandemic

Between 20th March and 10th December 2020, i.e., after the COVID-19 outbreak in the Czech Republic, we carried out the next wave of data collection, recruiting 353 pregnant women in collaboration with the General University Hospital in Prague. The procedure was similar to the preceding one: women were recruited during their prenatal medical check-ups at the hospital between the 11^th^ and 14^th^ gestational week, and they were asked to complete a questionnaire focusing on their sociodemographic background and health status and the DS-R and INVR questionnaires. Again, only healthy women who had conceived naturally were included in the study. Three women were excluded due to incomplete data for the DS-R (with more than one-fifth of items per each subscale or the whole questionnaire unanswered). In line with the stopping rule set in the preregistration, we completed the data collection when 350 participants were recruited during the pandemic. The final sample thus consisted of 350 pregnant women with complete data. The women were aged 20 to 44 years (see Table 1 for more details about the sample characteristics).

This research project has been approved by the Institutional Review Board at the Charles University, Faculty of Science (Approval No. 2020/07), and by the Ethics Committee of General University Hospital in Prague, Czech Republic (No. 384/16; 92/17). All participating women provided written informed consent.

### Measures

#### Disgust

The Disgust Scale-Revised (DS-R) (Olatunji et al., 2007) is a 25-item self-report inventory consisting of three subscales: Core disgust subscale (12 items; disgust elicited by food and animal or bodily products), Animal-reminder disgust subscale (8 items; disgust related to mortality, possible injuries, or violation of outer bodily envelope), and Contamination disgust subscale (5 items; disgust related to concerns about interpersonal transmission of pathogens). The items are rated on a 5-point scale ranging from 0 to 4. The overall DS-R score may thus range from 0 to 100, the score for the Core disgust subscale from 0 to 48, for the Animal-reminder subscale from 0 to 32, and for the Contamination disgust subscale from 0 to 20, with a higher score indicating greater disgust sensitivity. We used the Czech version of DS-R (Polák et al., 2019). If one-fifth or fewer responses were missing for each subscale, we used the average score of the corresponding subscale to supplement the missing values (we supplemented nine responses in the “before the pandemic” sample and five responses in the “during the pandemic” sample). The DS-R showed high internal consistency (before the pandemic: Cronbach’s alpha = 0.792, and during the pandemic: Cronbach’s alpha = 0.848). However, the internal consistency of the individual subscales was somewhat lower, with Cronbach’s alpha 0.656 and 0.726 for the Core disgust before and during the pandemic; 0.651 and 0.757 for the Animal-reminder disgust before and during the pandemic; and 0.415 and 0.564 for the Contamination disgust before and during the pandemic. Because of the unsatisfactory internal consistency of the Contamination disgust subscale and the factor structure analysis of the DS-R that did not support the three-factor model (3 subscales) in our data (see Preliminary analyses for more details), we only used the overall DS-R score in the main analyses. The results for the individual subscales are reported in the Supplementary materials.

#### Nausea and vomiting in pregnancy

The levels of NVP were assessed by the INVR (Rhodes and McDaniel, 1999). The INVR is a widely used instrument for assessing both intra-individual dynamics and inter-individual variation in NVP (Koken et al., 2008; Fiurašková et al., 2021). It consists of 8 items focusing on the symptoms that occurred in the worst form during the 12-hour period. There are five possible responses to each item (the score ranges from 0 to 4). The overall score (i.e., Rhodes Index) may thus range from 0 to 32, with a higher score indicating greater symptom severity. Participants with incomplete INVR questionnaires (with more than one-fifth of items unanswered) were excluded from the analyses (we excluded four women from the “before the pandemic” sample and six women from the “during the pandemic” sample). If one-fifth or fewer responses were missing, we used the average score for the questionnaire to supplement the missing values (we supplemented eight responses in the “before the pandemic” sample and eleven responses in the “during the pandemic” sample). The INVR showed high internal consistency in the samples of women recruited both before and during the pandemic (Cronbach’s alpha 0.831 and 0.830, respectively).

### Statistical analyses

Statistical analyses were performed using Jamovi 2.3.18 (Jamovi, 2022). As the Contamination subscale of the DS-R exhibited unsatisfactory internal consistency, we performed a confirmatory factor analysis (CFA) to test the factor structure of DS-R. We performed CFA for both the three-factor and the more parsimonious single-factor models suggested in the literature (Olatunji et al., 2007) using combined data from both samples and also data from each sample separately. Previous findings (Olatunji et al., 2014; Polák et al., 2019) also revealed that a bifactor model provided a good fit to DS-R data, suggesting that the measure is comprised of the general disgust factor while simultaneously including the Core, Animal-reminder, and Contamination disgust subscales. Therefore, we also performed CFA for the bifactor model (merging data from both samples).

We used ANCOVA with the independent binary variable “pandemic period” (before/during) and the DS-R and INVR scores as the dependent variable. Some variables (e.g., INVR score) showed deviation from the normal distribution (see Supplementary Table S1); however, the ANCOVA is robust with respect to such deviations, and we, therefore, report the results of the parametric tests. To assess the robustness of our findings, we also conducted analyses using the nonparametric partial Kendall correlations (the results are shown in the Supplementary materials). In line with the preregistration, we used one-sided tests to analyze the effects of the COVID-19 pandemic on disgust sensitivity (overall DS-R score and the Core and Contamination disgust subscales which are reported in the Supplementary materials). We controlled for maternal age in all models.

In line with the preregistration, we analyzed the effect of parity on disgust sensitivity depending on the pandemic, as several previous studies reported differences in disgust sensitivity in primiparae and multiparae, although with inconsistent findings (Żelaźniewicz and Pawłowski, 2015; Prokop and Fancovicova, 2016). In the preregistration, we planned to stratify the analyses for parity; however, we decided to test the effect of parity using an interaction term between the pandemic period and parity. We used ANCOVA with the independent variables “pandemic period,” parity and their interaction, and the total DS-R score as the dependent variable. We controlled for maternal age in all models.

## Results

### Preliminary analyses: Testing the DS-R factor structure

As the first step, we performed the CFA for the three-factor and single-factor models of DS-R structure using data merged from both samples and also data from each sample separately. The CFA indicated that the DS-R factor structure reported in previous studies (e.g., Olatunji et al., 2007; Żelaźniewicz and Pawłowski, 2015; Stevenson et al., 2021) is not supported by our data (Table 2). Subsequently, we tested the bifactor model with a general disgust factor, including simultaneously the Core, Animal-reminder, and Contamination disgust component factors. There were fitting issues caused by the independence of items 3 and 18 on the subfactors in the presence of the general disgust factor. It is customary to use the bifactor model, where the problematic items load only on the general factor. Therefore, we removed the loadings of item 3 on the Core and item 18 on the Contamination disgust subscales and let them load only on the general disgust factor. The bifactor model showed the best fit (Table 2). Based on the hierarchical omega for that model (0.83, 95% CI = 0.81–0.85), we decided to use the overall disgust score and not the subscale scores for the main analysis in this study. The results for the individual subscales are reported in the Supplementary materials.

**Table 2:** Comparison of different models of the Czech DS-R using a confirmatory factor analysis.

	*χ*2	df	RMSEA	RMSEA 90%CI	CFI	TLI
Both samples						
1-factor model	870**	275	0.063	0.058–0.068	0.746	0.723
3-factor model	696**	272	0.053	0.048–0.058	0.819	0.800
Bifactor model	530**	250	0.045	0.040–0.050	0.966	0.959
Sample 1						
1-factor model	480**	275	0.061	0.052–0.070	0.688	0.660
3-factor model	443**	272	0.056	0.046–0.065	0.074	0.713
Sample 2						
1-factor model	723**	275	0.068	0.062–0.074	0.743	0.720
3-factor model	579**	272	0.057	0.050–0.063	0.824	0.806

**χ2 test is significant at *p* < 0.001, df degrees of freedom, RMSEA root mean square error of approximation, CFI comparative fit index, TLI Tucker-Lewis index; bifactor model after removing the loading of items 3 and 18 on the subscales.

Despite the good RMSEA (0.045) and acceptable CFI (0.966) for the bifactor model, we identified two problematic items (3, 18) in this model. Therefore, as the follow-up analyses, we also performed the exploratory factor analysis (EFA). A parallel analysis with oblimin rotation using the maximum likelihood method suggested up to 6 inter-correlated factors (see Supplementary Table S2). While we identified the Animal-reminder factor (subscale), other factors tended to mix the elements from the original Core and Contamination disgust subscales. Since the single-factor model – unlike the models with several factors – was supported by our data, we also performed EFA for the single-factor model. The EFA results indicated that items 4, 6, and 10 should be omitted as they had loadings onto the single factor < 0.3. We therefore removed those items from the overall questionnaire score which we calculated using 10 Berge estimation method. We report the results for this new version of the questionnaire labeled DS-R-22 in the (see Supplementary Tables S1, S3, S4, S5).

### Disgust sensitivity in pregnant women before and during the COVID-19 pandemic

The mean scores of the DS-R for both samples (recruited before and during the pandemic) are shown in Table 3 (for the DS-R subscales see Supplementary Table S3). The analyses of covariance (ANCOVA) with age as a covariate (*F*_1,547_ = 3.60, *p* = 0.058) showed that women who were pregnant during the COVID-19 pandemic had higher disgust sensitivity compared to those who were pregnant before the pandemic (*F*_1,547_ = 3.87, *p* = 0.025 and *F*_1,545_ = 3.04, *p* = 0.041, respectively) (Table 3, results for the DS-R subscales and DS-R-22 are shown in Supplementary Table S3, for nonparametric tests see Supplementary Table S4).

**Table 3:** Disgust sensitivity (DS-R) and nausea and vomiting (INVR) in pregnant women before and during the COVID-19 pandemic.

	Before pandemic			During pandemic			Statistical models		
	*N*	Mean	SD	*N*	Mean	SD	*F*	*p*	Cohen’s *d*
Total disgust	200	50.5	13.1	350	52.7	15.0	3.87	0.025	0.18
Nausea and vomiting	196	9.29	6.59	344	8.80	6.43	0.35	0.553	0.05

ANCOVA controlling for maternal age; Cohen’s d effect size. In line with the preregistration, one-sided test was used for total disgust.

### Effect of parity on disgust sensitivity in pregnant women before and during the COVID-19 pandemic

**Figure 1:**
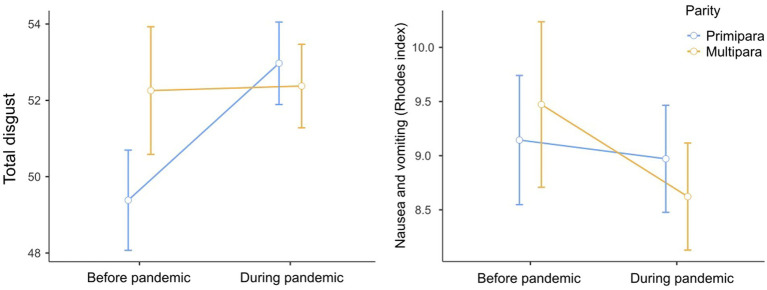
Disgust sensitivity and nausea and vomiting (mean, SE) in pregnant women before and during the COVID-19 pandemic — Stratified by parity.

**Figure 2:**
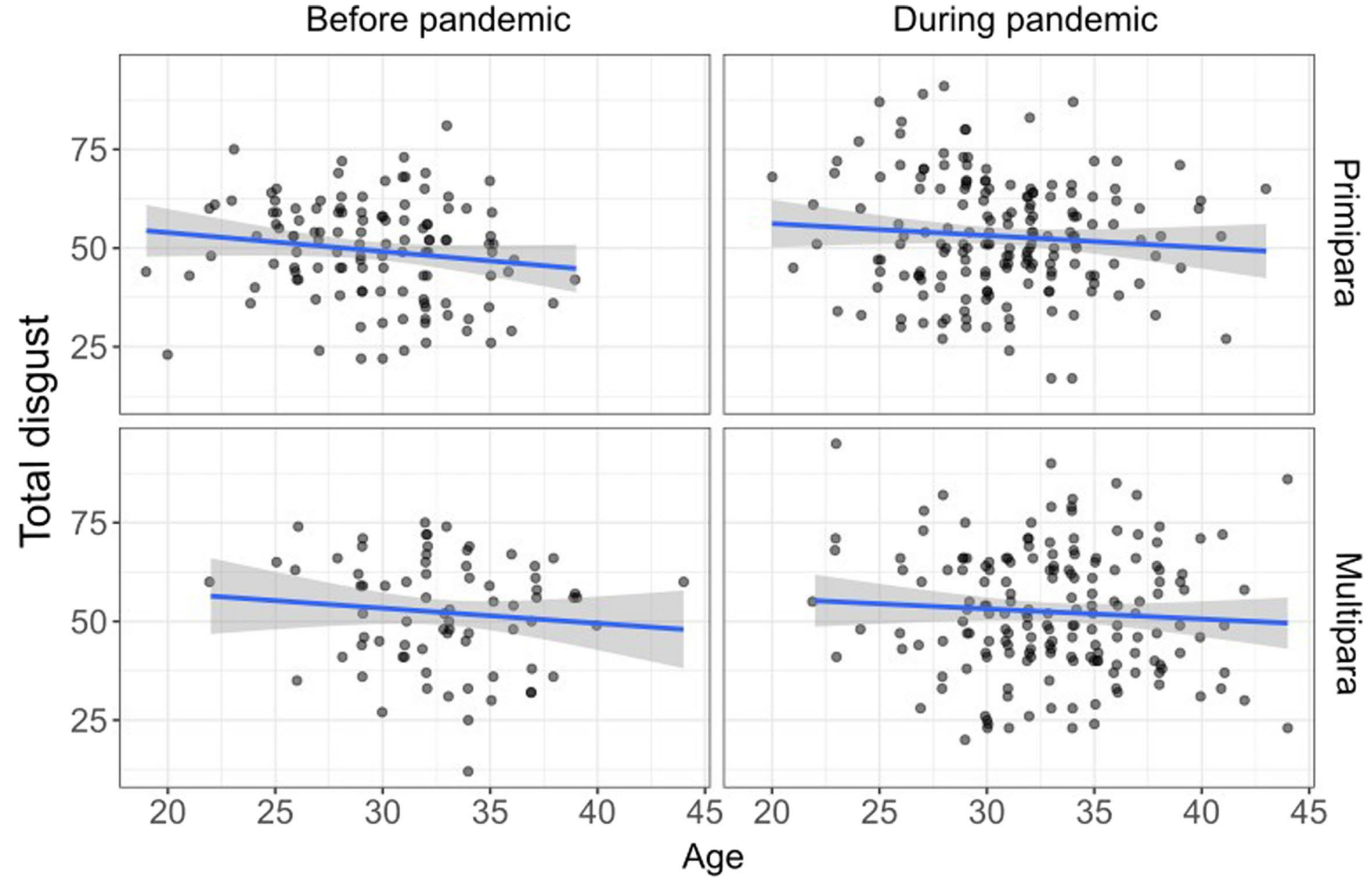
Effect of age on disgust sensitivity in pregnant women before and during the COVID-19 pandemic — Stratified by parity (fitted line for regression, confidence bands).

The mean scores on the DS-R calculated separately for primiparae and multiparae are shown in Figure 1 and Table 4 (for the mean DS-R subscales scores see Supplementary Figure S1). The analysis (ANCOVA) of the pandemic and parity on disgust sensitivity (controlling for age as a covariate) showed no significant effects of the pandemic (*F*_1,539_ = 2.55, *p* = 0.056), parity (*F*_1,539_ = 2.32, *p* = 0.128) or their interaction (*F*_1,539_ = 2.08, *p* = 0.150) (for the results related to the individual DS-R subscales and DS-R-22 see Supplementary Table S5). In this model, only the effect of age was significant (*F*_1,539_ = 4.77, *p* = 0.029), indicating that disgust sensitivity decreased with age (Figure 2).

**Table 4:** The means of total disgust sensitivity scale (DS-R) before and during the pandemic separately for primiparous and multiparous women.

		*N*	Mean	SD
Primiparous women	Before pandemic	120	49.4	12.7
	During pandemic	177	53.0	14.4
Multiparous women	Before pandemic	74	52.3	13.8
	During pandemic	173	52.4	15.6

Nevertheless, as the visual inspection of the data indicated that there might be differences in disgust sensitivity between primiparous and multiparous women (Figure 1), and also in line with the preregistration planning to stratify the analyses for parity, we also performed *post hoc* comparisons for parity and the pandemic period (Table 5). These comparisons showed significantly lower disgust sensitivity in primiparous women recruited before the pandemic compared to both primiparous (*t*_539_ = −2.34, *p* = 0.020) and multiparous (*t*_539_ = −2.35, *p* = 0.019) women recruited during the pandemic. To account for the possible effect of age, we repeated the ANCOVA with age as a covariate assessing the effect of the pandemic on the overall DS-R score in primiparous women only. This analysis showed a significant effect of the pandemic on disgust sensitivity in primiparae (*F*_1,294_ = 6.05, *p* = 0.007, Cohen’s *d* = 0.29).

**Table 5:** *Post hoc* comparisons — The effect of the pandemic and parity on disgust sensitivity (overall DS-R score) in pregnant women.

Comparison									
Pandemic	Parity		Pandemic	Parity	Mean difference	SE	df	*t*	*p*
1	0	–	1	1	−3.99	2.18	539	−1.83	0.068
		–	2	0	−3.98	1.70	539	−2.34	0.020
		–	2	1	−4.20	1.79	539	−2.35	0.019
	1	–	2	0	0.01	2.01	539	0.004	0.997
		–	2	1	−0.21	1.99	539	−0.10	0.917
2	0	–	2	1	−0.22	1.58	539	−0.14	0.891

Comparisons are based on estimated marginal means; Pandemic: 1 = before pandemic, 2 = during pandemic; Parity: 0 = primiparae, 1 = multiparae. Each row compares two different groups of women depending on Pandemic and Parity. The first group of women is defined by columns 1 and 2, and the second group of women by columns 4 and 5.

### Nausea and vomiting in pregnant women before and during the COVID-19 pandemic

**Figure 3:**
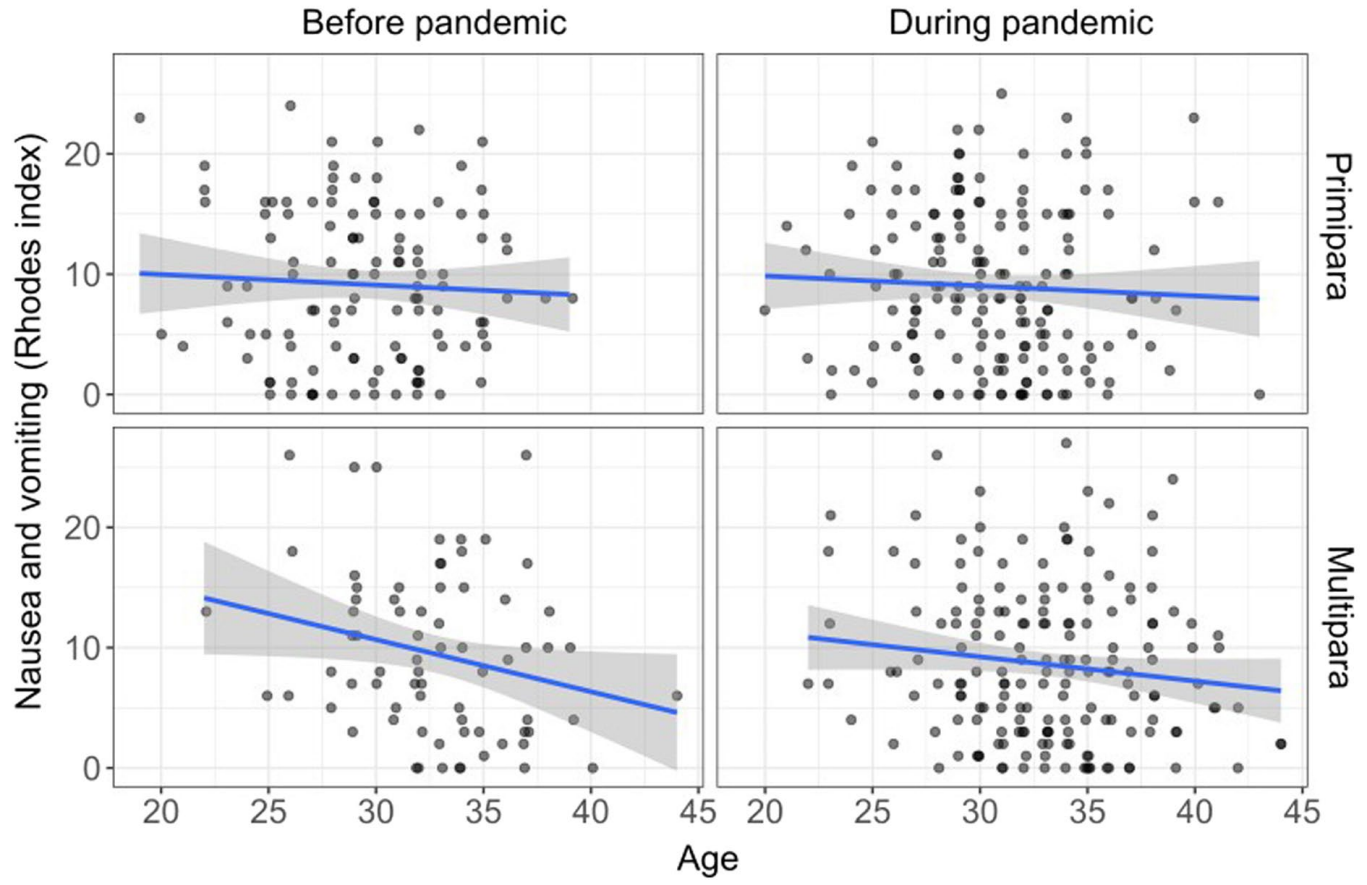
Effect of age on nausea and vomiting in pregnant women before and during the COVID-19 pandemic — Stratified by parity (fitted line for regression, confidence bands).

The analyses of covariance with age as a covariate (*F*_1,537_ = 3.61, *p* = 0.058) showed no significant differences between the women recruited before and during the pandemic in terms of NVP (*F*_1,537_ = 0.35, *p* = 0.553) (Table 3). After adding parity to the analyses, we also found no significant effects of parity (*F*_1,529_ = 0.57, *p* = 0.451), pandemic (*F*_1,529_ = 0.43, *p* = 0.515), and their interaction (*F*_1,529_ = 0.47, *p* = 0.493) on NVP in the model adjusted for maternal age. In this analysis, we found a significant effect of age on NVP (*F*_1,529_ = 5.89, *p* = 0.016), such that older women experienced less NVP (Figure 3).

## Discussion

In this study, we examined differences in pregnant women’s disgust sensitivity in the pre-pandemic versus the COVID-19 pandemic period. We pre-registered a hypothesis that women who were pregnant during the pandemic would display higher scores on the Disgust Scale-Revised (DS-R) compared to those who were pregnant before the pandemic. This hypothesis is supported by our data, as we found that women who were pregnant during the pandemic versus those pregnant before the pandemic showed indeed statistically significantly higher disgust sensitivity (Cohen’s *d* = 0.18). The interaction between the pandemic period and parity was not significant, however, in the follow-up analyses, we found significant differences in disgust sensitivity the pre-pandemic and during the pandemic periods in primiparae only (Cohen’s *d* = 0.29). In line with the preregistration, we found no statistically significant differences in intensity of nausea and vomiting in pregnancy between the women pregnant before and during the pandemic (Cohen’s *d* = 0.05).

### The effect of the COVID-19 pandemic on disgust sensitivity

Our finding that disgust increased with the COVID-19 outbreak is in line with previous studies reporting higher levels of disgust sensitivity during the COVID-19 lockdown, including an Australian study comparing disgust sensitivity in students during the pandemic and in the period 2008–2010 (Stevenson et al., 2021). Another study found elevated pathogen disgust during the pandemic in a sample of Polish women, but only when using disgust-evoking images; no difference in disgust was found when disgust was measured with the Three Domain Disgust Scale (Miłkowska et al., 2021b).

However, it should be pointed out that the effect of the pandemic on disgust sensitivity observed in our study is rather small (Cohen’s *d* = 0.18). The reason for such a small effect could be that pregnant women, especially in the first trimester of pregnancy, may have elevated disgust sensitivity due to pregnancy itself (Fessler et al., 2005; Żelaźniewicz and Pawłowski, 2015). Pregnancy is characterized by intensive immunomodulation, which may lead to a higher need for behavioral protection of the fetus (Kaňková et al., 2022). As BIS (including disgust sensitivity) is already activated in pregnancy, we may presume that the higher risk of contracting infection during the pandemic affects pregnant women’s disgust sensitivity only to a limited extent. Indeed, pregnant women showed a higher mean overall DS-R score (mean = 50.5, SD = 13.1) in our sample collected before the pandemic compared to non-pregnant 679 Czech women, predominantly high school or college students, from the study by Polák et al. (2019) conducted before the pandemic outbreak (mean = 46.06, SD = 14.07).

Nevertheless, we should exercise caution while exploring the role of BIS in the context of the COVID-19 pandemic. Despite some similarities between the severe acute respiratory syndrome coronavirus 2 (SARS-CoV-2) and the more common infectious diseases in terms of symptoms and modes of transmission, the psychological response to the pandemic might be guided by other mechanisms than the pathogen-avoiding response to the more common, non-pandemic infectious diseases, that has been shaped in the evolution of the BIS (Ackerman et al., 2021).

### The effect of interaction between the pandemic and parity on disgust sensitivity

Contrary to our expectations, we found no interaction between the pandemic period and parity in their effect on disgust sensitivity. This may be the result of a limited power and it is possible that such interaction would be significant in a larger sample. Indeed, it has recently been shown (Blake and Gangestad, 2020) that to detect an attenuated interaction, as in this case, much larger samples are needed to obtain sufficient power. Interestingly, the results of the post-hoc analysis indicate that the increase in disgust sensitivity as a response to the COVID-19 pandemic applies particularly to primiparous women. Primiparous women’s disgust sensitivity before the pandemic was significantly lower compared to primiparae during the pandemic, a finding that did not apply to multiparae. Moreover, we found lower disgust sensitivity in primiparae before the pandemic compared to multiparae during the pandemic. It is possible that women caring for young children generally display higher disgust sensitivity compared to childless women. The higher disgust sensitivity in mothers might be essential so they can teach their children how to avoid potential pathogens and other health-related risks (Al-Shawaf et al., 2018) as children learn to a great extent through imitation (Stevenson et al., 2010; Muris et al., 2013). However, Prokop and Fancovicova (2016) found lower disgust sensitivity in mothers compared to childless women.

A possible mechanism of the differences in disgust sensitivity depending on parity may lie in changes in progesterone levels. As noted above, a higher level of progesterone has been associated with increased disgust sensitivity (Fleischman and Fessler, 2011; Żelaźniewicz et al., 2016; Miłkowska et al., 2021a). We could therefore assume that multiparous women have higher levels of progesterone which leads to an increase in disgust sensitivity independently of the pandemic (see Figure 1). However, previous research found no differences in progesterone levels between primiparous and multiparous women (Wuu et al., 2002; Lof et al., 2009; Grossi et al., 2019) and Toriola et al. (2011) even found lower levels of progesterone in multiparae compared to primiparae.

### Disgust sensitivity and maternal age

Our results also show that disgust sensitivity decreases with maternal age (from 19 to 44 years). This is in line with the results of previous studies showing a negative effect of age on disgust sensitivity in non-pregnant women of a similar age range (Fessler and Navarrete, 2003) and both men and women aged 16 to 89 years (Polák et al., 2019). In contrast, a recent study (Díaz et al., 2020) found a positive association between disgust sensitivity and age in participants aged 18–64 years. Generally, the vulnerability to diseases increases with age because of the age-related decline in the functionality of the physiological immune system. It was therefore argued that such a decline could be compensated by an increased disgust sensitivity in older people (Oaten et al., 2009).

### The DS-R scale and its factor structure—results for the subscales

We originally aimed to use the total DS-R score along with the individual DS-R subscale scores as the outcome variables. Nevertheless, due to the low internal consistency of the Contamination disgust subscale and based on the results of the CFA that did not support the previously reported three-factor DS-R structure (Olatunji et al., 2007), we decided to present only the results for the overall DS-R score in the main analyses, while adding the results for the individual subscales to the Supplementary materials. The results for the individual subscales showed that pregnant women experienced higher Contamination disgust (i.e., disgust related to concerns about interpersonal transmission of pathogens) during the pandemic compared to the pre-pandemic period (Supplementary Table S3). Our study thus provides evidence that disgust sensitivity increases adaptively when individuals face a higher risk of infection through interpersonal contact. Consistent with our findings, Miłkowska et al. (2021b) reported that women scored higher on the Contamination Obsession and Washing Compulsion Subscale of Padua Inventory during the pandemic. Moreover, several other studies have linked the COVID-19 pandemic to enhanced hygiene and safety behavior, such as hand washing (Korajlija and Jokic-Begic, 2020; Stevenson et al., 2021). However, as the internal consistency of the Contamination subscale was unsatisfactory, these results should be interpreted with caution. We found no statistically significant association between the COVID-19 pandemic and the Core disgust subscale when comparing the women pregnant before and during the pandemic. Contrary to our results, using the same questionnaire as in the present study (DS-R), Stevenson et al. (2021) showed that during the COVID-19 pandemic, students had higher scores in the Core disgust subscale compared to the pre-pandemic period.

In the Supplementary materials, we also present the results for the DS-R-22 version which is based on the EFA with our data. The analyses for DS-R and DS-R-22 provided similar results, suggesting robustness of our findings.

### Association between the COVID-19 pandemic and nausea and vomiting in pregnancy

We found no significant differences in the frequency of nausea and vomiting during and before the COVID-19 pandemic, which is in line with our preregistered hypothesis. One of the possible functions of nausea in pregnancy is, similarly to the function of disgust, to protect the fetus and the mother against potentially harmful substances (Hook, 1976; Profet, 1992, 1995; Flaxman and Sherman, 2000). It is assumed that nausea and vomiting in pregnancy lead to the avoidance of food containing potentially toxic abortifacients and teratogens, such as alcohol, caffeine, and tobacco, but also animal products, such as meat, fish, eggs, and milk, probably because these foods are quickly perishable (Hook, 1976; Flaxman and Sherman, 2000; Fiurašková et al., 2021). Moreover, nausea and vomiting in pregnancy may also be elicited by specific plants, such as pungent or bitter vegetables and herbs, that are rich in toxic phytochemicals (Profet, 1992, 1995). Despite the similar function of disgust sensitivity and nausea and vomiting in pregnancy, our results indicate that they involve distinct mechanisms. Proximate causes of nausea and vomiting in pregnancy primarily include physiological changes generally related to pregnancy, leading to avoiding harmful foods, mainly because of their toxicity, whereas disgust sensitivity also reflects the actual level of the pathogen threat in the surrounding environment. Consistent with other evidence (Chortatos et al., 2013; Dekkers et al., 2020), and similarly to disgust sensitivity, we observed that younger women had increased severity and incidence of NVP than older women.

## Limitations

The main limitation of this study is that it is based on the between-subject comparison using the longitudinal trend design, while longitudinal design would be more appropriate to determine the associations between the onset of the COVID-19 pandemic and disgust sensitivity. Nevertheless, it is impossible to obtain data on disgust sensitivity before and during the pandemic from the same sample of pregnant women at the same phase of pregnancy. To minimize the potential effect of inter-individual differences, we collected data at the same maternity hospital at the same pregnancy phase in both data collection waves.

Another potential limitation concerns the method we used to assess disgust sensitivity in pregnant women. While we used the DS-R questionnaire to be able to compare our results with the existing studies on disgust sensitivity in pregnancy (Fessler et al., 2005; Żelaźniewicz and Pawłowski, 2015), it would be more appropriate to use a method developed within an evolutionary framework, such as the Three Domains of Disgust Scale (Tybur et al., 2009), when examining the role of disgust in the context of the Compensatory Prophylaxis Hypothesis. Another disgust scale that fits into the BIS framework is the Body Odor Disgust Scale (Liuzza et al., 2017), which was not yet used in pregnant women.

Moreover, although we found differences in disgust sensitivity in pregnancy depending on the pandemic threat, we cannot address the question regarding the psychological mechanisms responsible for this effect based on our questionnaire data. This effect can be attributed to the higher sensitivity to the disgust-related cues, or such cues can be interpreted as disgusting only during the higher-risk period. Of course, such low- and high-level cognitive mechanisms are not mutually exclusive and may in fact work in concert. Future studies should go beyond survey methods as was used here and employ exposure to disgust-eliciting stimuli or behavioral tests.

Additionally, as data collection took place in a hospital environment, it could be argued that the observed differences in disgust levels in the pandemic versus pre-pandemic period are due to increased prophylactic behavior of pregnant women who might have been concerned about contracting COVID-19 in the higher-risk hospital environment. However, we believe that this is not the case, as the data were collected at the maternity hospital of the General University Hospital in Prague (Dept. of Obstetrics and Gynecology), which resides in a separate building, where only obstetric (including newborn infants) and gynecological care is provided which limits the exposure to the disease-related cues as compared to regular hospitals. With strict measures in place during the COVID-19 pandemic including banned entry for anyone except for the patients and personnel, the risk of contracting COVID-19 was very low.

## Conclusion

This preregistered study provides novel evidence of how pregnant women’s disgust sensitivity adjusts adaptively depending on the actual environmental pathogen risks. Although there was higher disgust sensitivity in pregnant women during versus before the COVID-19 pandemic, this effect was rather weak. We suggest that in pregnant women, disgust elevates only slightly as a response to a higher risk of infection during the pandemic, as it is already elevated due to pregnancy itself. Although there was no effect of interaction between parity and the pandemic period on disgust, a slightly stronger effect of the pandemic on disgust was found in the subset of primiparous women, suggesting that a prior pregnancy experience could play a role in the BIS activation during pregnancy. We found no differences in terms of nausea and vomiting between the women pregnant before and during the pandemic. These findings indicate that although BIS has evolved as a complex mechanism to prevent health-threatening behaviors, its activation is rather weak in the context of the COVID-19 pandemic in pregnant women.

## Data availability statement

The dataset presented in this study can be found in online repositories. The names of the repository/repositories and accession number(s) can be found at: https://doi.org/10.6084/m9.figshare.16843210.v1, repository name “figshare.”

## Ethics statement

The studies involving human participants were reviewed and approved by Ethical Committee for Human Research of the Faculty of Science Charles University and Ethical Committee of General University Hospital in Czech Republic. The patients/participants provided their written informed consent to participate in this study.

## Author contributions

ŠK, LT, CM, and JHa developed the study concept. PC recruited participants. JHl, ŠK, and PC performed data collection. ŠK conducted data analysis. ŠK, LT, and JHa drafted the manuscript. All authors contributed to the study design and participated in editing and revising the manuscript.

## Funding

This work was supported by the Czech Science Foundation project GAČR 20-16698S “Disgust sensitivity in pregnancy: Individual differences and longitudinal changes” (ŠK, LT, JHa, and JHl). This work was also supported by the European Regional Development Fund –Project “Creativity and Adaptability as Conditions of the Success of Europe in an Interrelated World” (No. CZ.02.1.01/0.0/0.0/16_019/0000734) (LT), by Charles University Research Centre program No. 204056 (ŠK, JHa) and the Ministry of Health of the Czech Republic (grant RVO-VFN64165) (PC).

## Conflict of interest

The authors declare that the research was conducted in the absence of any commercial or financial relationships that could be construed as a potential conflict of interest.

## Publisher’s note

All claims expressed in this article are solely those of the authors and do not necessarily represent those of their affiliated organizations, or those of the publisher, the editors and the reviewers. Any product that may be evaluated in this article, or claim that may be made by its manufacturer, is not guaranteed or endorsed by the publisher.

## Footnotes

^1^https://osf.io/bmhku/
