# Supplementary material for: Disgust sensitivity in early pregnancy as a response to high pathogen risk
Source: Front Psychol. 2023 Feb 27;14:1015927. doi: 10.3389/fpsyg.2023.1015927 (PMC10009253; doi:10.3389/fpsyg.2023.1015927)
Supplement: Supplementary file 1 [file Data_Sheet_1.docx]

Supplementary Materials

**Disgust sensitivity in early pregnancy as a response to high pathogen risk**

**Table S1**

Analyses of data distribution

|  |  | **Statistical models** | | | | | |
| --- | --- | --- | --- | --- | --- | --- | --- |
|  |  | **All** | | **Primiparous** | | **Multiparous** | |
|  |  | p | Shapiro-Wilk W | p | Shapiro-Wilk W | p | Shapiro-Wilk W |
| Before pandemic | Total disgust | .080 | 0.988 | .260 | 0.986 | .073 | 0.970 |
|  | Core | .013 | 0.982 | .179 | 0.984 | .007 | 0.952 |
|  | Contamination | .021 | 0.984 | .055 | 0.979 | .394 | 0.982 |
|  | Animal-reminder | .185 | 0.990 | .085 | 0.981 | .503 | 0.985 |
|  | DS-R-22 | .002 | 0.983 | .389 | 0.988 | .009 | 0.953 |
|  | Nausea and vomiting | < .001 | 0.956 | < .001 | 0.954 | .005 | 0.948 |
| During pandemic | Total disgust | .116 | 0.993 | .506 | 0.993 | .170 | 0.988 |
|  | Core | .027 | 0.991 | .259 | 0.990 | .079 | 0.986 |
|  | Contamination | < .001 | 0.979 | .003 | 0.975 | .010 | 0.979 |
|  | Animal-reminder | .010 | 0.989 | .517 | 0.993 | .021 | 0.981 |
|  | DS-R-22 | .135 | 0.993 | .590 | 0.993 | .141 | 0.998 |
|  | Nausea and vomiting | < .001 | 0.953 | < .001 | 0.952 | < .001 | 0.948 |

**Table S2**

Factor loading of the disgust elicitor items form DS-R (subscales: Core, Animal-reminder, Contamination)

|  | | **Factor** | | | | | | | | | | | |  | |
| --- | --- | --- | --- | --- | --- | --- | --- | --- | --- | --- | --- | --- | --- | --- | --- |
| **Item - Subscale** | | **1** | | **2** | | **3** | | **4** | | **5** | | **6** | | **Uniqueness** | |
| 1 - Core |  | 0.042 |  | 0.171 |  | 0.025 |  | 0.081 |  | -0.220 |  | **0.302** |  | 0.797 |  |
| 3 - Core |  | -0.024 |  | 0.115 |  | -0.035 |  | 0.078 |  | **0.606** |  | 0.059 |  | 0.555 |  |
| 6 - Core |  | 0.001 |  | 0.187 |  | 0.048 |  | -0.010 |  | -0.037 |  | -0.003 |  | 0.960 |  |
| 8 - Core |  | 0.197 |  | 0.018 |  | 0.203 |  | 0.005 |  | **0.473** |  | -0.141 |  | 0.664 |  |
| 11 - Core |  | 0.042 |  | **0.509** |  | -0.166 |  | 0.032 |  | -0.022 |  | 0.048 |  | 0.736 |  |
| 13 - Core |  | 0.049 |  | 0.027 |  | 0.130 |  | 0.021 |  | 0.008 |  | **0.536** |  | 0.632 |  |
| 15 - Core |  | -0.034 |  | **0.557** |  | 0.068 |  | -0.002 |  | 0.155 |  | 0.015 |  | 0.605 |  |
| 17 - Core |  | -0.077 |  | **0.337** |  | 0.200 |  | 0.090 |  | 0.064 |  | 0.180 |  | 0.685 |  |
| 20 - Core |  | 0.080 |  | 0.162 |  | **0.345** |  | 0.156 |  | -0.021 |  | 0.075 |  | 0.676 |  |
| 22 - Core |  | 0.024 |  | 0.077 |  | **0.416** |  | 0.030 |  | 0.063 |  | 0.253 |  | 0.654 |  |
| 25 - Core |  | -0.007 |  | 0.019 |  | **0.659** |  | 0.011 |  | 0.029 |  | 0.027 |  | 0.546 |  |
| 27 - Core |  | 0.150 |  | **0.454** |  | 0.111 |  | 0.006 |  | -0.038 |  | -0.108 |  | 0.684 |  |
| 2 - An-r |  | **0.339** |  | 0.142 |  | -0.269 |  | 0.020 |  | 0.143 |  | 0.175 |  | 0.673 |  |
| 5 – An-r |  | 0.110 |  | 0.161 |  | 0.041 |  | 0.063 |  | 0.046 |  | 0.095 |  | 0.882 |  |
| 7 – An-r |  | **0.633** |  | -0.013 |  | -0.111 |  | 0.035 |  | 0.132 |  | 0.178 |  | 0.451 |  |
| 10 – An-r |  | 0.166 |  | 0.170 |  | -0.173 |  | -0.009 |  | 0.052 |  | 0.010 |  | 0.908 |  |
| 14 – An-r |  | **0.338** |  | 0.060 |  | 0.053 |  | -0.015 |  | 0.075 |  | **0.330** |  | 0.638 |  |
| 19 – An-r |  | **0.593** |  | 0.051 |  | 0.054 |  | 0.169 |  | -0.160 |  | -0.107 |  | 0.565 |  |
| 21- An-r |  | **0.594** |  | 0.090 |  | 0.045 |  | -0.116 |  | 0.142 |  | -0.085 |  | 0.571 |  |
| 24 – An-r |  | **0.483** |  | 0.079 |  | 0.216 |  | 0.038 |  | -0.129 |  | 0.144 |  | 0.553 |  |
| 4 - Cont |  | 0.038 |  | -0.121 |  | -0.025 |  | 0.176 |  | 0.293 |  | 0.216 |  | 0.811 |  |
| 9 - Cont |  | -3.78e^−4^ |  | 0.171 |  | -0.002 |  | 0.174 |  | 0.171 |  | 0.062 |  | 0.851 |  |
| 18 - Cont |  | -0.090 |  | -0.012 |  | -0.009 |  | **0.851** |  | 0.030 |  | -0.010 |  | 0.292 |  |
| 23 - Cont |  | 0.072 |  | 0.179 |  | 0.240 |  | 0.210 |  | -0.005 |  | 0.074 |  | 0.717 |  |
| 26 - Cont |  | 0.103 |  | 0.237 |  | 0.169 |  | 0.281 |  | -0.119 |  | -0.034 |  | 0.692 |  |
| The maximum likelihood' extraction method was used in combination with 'oblimin' rotation | | | | | | | | | | | | | | | |

**Table S3**

Disgust sensitivity (Subscales of DS-R and DS-R-22 based on EFA) in pregnant women before and during the COVID-19 pandemic

|  | **Before pandemic** | | | **During pandemic** | | | **Statistical models** | | |
| --- | --- | --- | --- | --- | --- | --- | --- | --- | --- |
|  | **N** | **Mean** | **SD** | **N** | **Mean** | **SD** | **F** | **p** | **Cohen’s d** |
| Core | 200 | 26.3 | 7.08 | 350 | 27.2 | 7.76 | 2.36 | .063 | 0.14 |
| Contamination | 200 | 7.22 | 3.16 | 350 | 8.09 | 3.54 | 8.13 | <.001 | 0.25 |
| Animal-reminder | 200 | 17.0 | 5.78 | 350 | 17.4 | 6.63 | 1.18 | .277 | 0.10 |
| DS-R-22 | 198 | -0.08 | 0.84 | 350 | 0.04 | 0.97 | 3.04 | .041 | 0.16 |

ANCOVA controlling for maternal age; Cohen’s d effect size

In line with the pre-registration, one-sided test was used for Core and Contamination disgust variables

**Table S4**

Differences in disgust sensitivity (DS-R) before and during the pandemic using nonparametric tests.

|  | **Statistical models** | | |
| --- | --- | --- | --- |
|  | p | Tau B | Cohen's d |
| Total disgust | .034 | 0.052 | 0.17 |
| Core | .133 | 0.032 | 0.10 |
| Contamination | < .001 | 0.091 | 0.29 |
| Animal-reminder | .164 | 0.040 | 0.13 |
| DS-R-22 | .061 | 0.044 | 0.14 |
| Nausea and vomiting | .408 | -0.024 | 0.08 |

Partial Kendall correlation was used, adjusted for maternal age

In accordance with the preregistration, one-sided test was used for both Total disgust, Core and Contamination disgust scales

**Table S5**

The effect of interaction between the pandemic and parity on disgust sensitivity (subscales of DS-R and DR-R-22 based on EFA) in pregnant women

|  |  | Core | | | Contamination | | Animal-reminder | | DS-R-22 | |  |
| --- | --- | --- | --- | --- | --- | --- | --- | --- | --- | --- | --- |
|  |  | F | | p | F | p | F | p | F | p |  |
| Age | | | 3.46 | .064 | 0.09 | .770 | 6.66 | .010 | 6.50 | .011 | |
| Pandemic | | | 1.64 | .101 | 7.33 | .004 | 0.40 | .528 | 1.85 | .087 | |
| Parity | | | 0.30 | .587 | 1.56 | .213 | 4.56 | .033 | 2.82 | .094 | |
| Pandemic  *Parity | | | 1.92 | .167 | 0.56 | .814 | 2.22 | .137 | 1.88 | .170 | |

In line with the preregistration, one-sided test was used for the effects of the pandemic on Core and Contamination disgust
