# Supplementary figures and images for: Disgust sensitivity in early pregnancy as a response to high pathogen risk
Source: Front Psychol. 2023 Feb 27;14:1015927. doi: 10.3389/fpsyg.2023.1015927 (PMC10009253; doi:10.3389/fpsyg.2023.1015927)

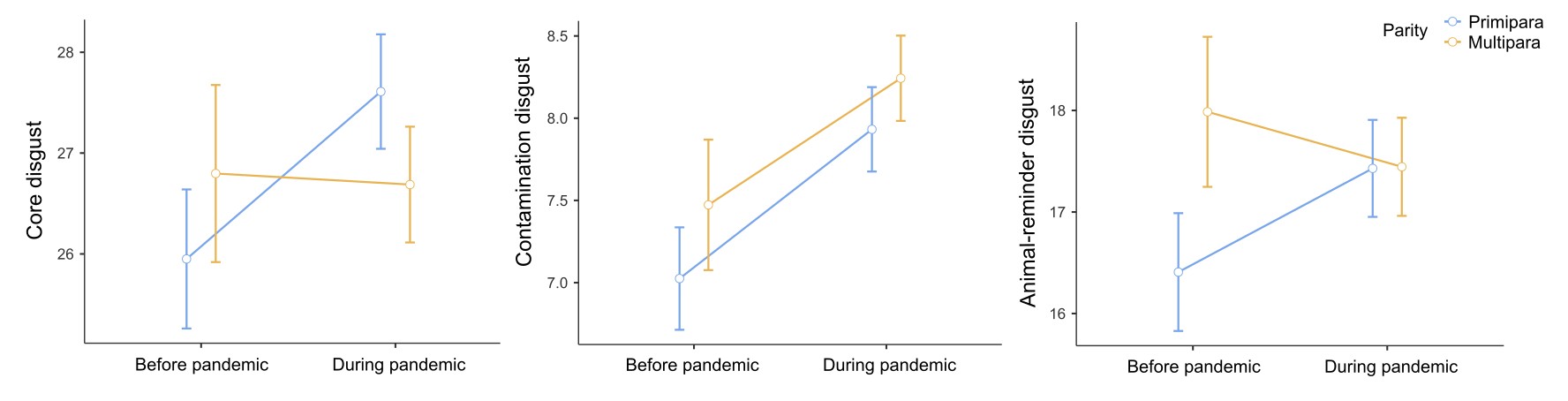

Supplement: Supplementary file 2 [file Image_1.JPEG]
